# The epidemiology of porcine *Taenia solium* cysticercosis in communities of the Central Highlands in Vietnam

**DOI:** 10.1186/s13071-018-2945-y

**Published:** 2018-06-22

**Authors:** Dinh Ng-Nguyen, John Noh, Kathleen Breen, Mark Anthony Stevenson, Sukwan Handali, Rebecca Justine Traub

**Affiliations:** 1 Faculty of Veterinary and Agricultural Sciences, University of Melbourne, Parkville, Victoria 3052 Australia; 2 Faculty of Animal Sciences and Veterinary Medicine, Tay Nguyen University, Buon Ma Thuot, Dak Lak, Vietnam; 3 Division of Parasitic Diseases and Malaria, Centers for Disease Control and Prevention, Atlanta, Georgia USA; 4 Department of Livestock, Montana Veterinary Diagnostic Lab, Bozeman, Montana USA

**Keywords:** *Taenia solium*, Porcine cysticercosis, Epidemiology, Vietnam

## Abstract

**Background:**

*Taenia solium* cysticercosis, recognized as a neglected tropical disease by the WHO, is distributed mostly in developing countries of Latin America, sub-Saharan Africa and Asia. Pigs and humans act as intermediate hosts, acquiring *T. solium* cysticerci (larval stage) in their tissue, through the ingestion of *T. solium* eggs shed in the faeces of humans infected with adult tapeworms. The disease has a negative impact on rural economies due to losses in productivity arising from human disease, pork carcass condemnations and loss of market access. The aim of this study was to estimate the prevalence of *T. solium* cysticercosis in pigs in Dak Lak Province in the Central Highlands of Vietnam and to identify household level characteristics associated with *T. solium* porcine cysticercosis.

**Methods:**

This was a cross-sectional study of household pigs in three districts of Dak Lak Province. A total of 408 households in six villages in three districts were visited between June and October 2015. A questionnaire was administered to the head of each household, and within each household, serum samples were collected from three pigs. Serum samples were analyzed using the recombinant T24H antigen in enzyme-linked immunoelectrotransfer blot assay and lentil lectin purified glycoprotein in EITB assay. A Bayesian, mixed-effects logistic regression model was developed to identify management factors associated with the probability of a household having at least one cysticercosis-positive pig.

**Results:**

The prevalence of porcine *T. solium* cysticercosis in this study was low at 0.94 [95% confidence interval (CI) 0.51–1.68] cases per 100 pigs at risk, in agreement with other studies conducted throughout Vietnam. Scavenging of food and coprophagy were associated with *T. solium* cysticercosis [odds ratios 1.98 (95% CrI: 0.55–4.74) and 2.57 (95% CrI: 1.22–4.66), respectively].

**Conclusions:**

This study proves that the seroprevalence of porcine cysticercosis in Dak Lak Province was as low as that of other studies conducted throughout Vietnam. Scavenging of food and coprophagy are modifiable factors, providing the opportunity to decrease the prevalence of porcine cysticercosis further in the province.

## Background

*Taenia solium* cysticercosis is recognized as a neglected tropical disease by the WHO [1]. It is distributed mostly in developing countries of Latin America, sub-Saharan Africa and Asia [2]. Pigs and humans act as intermediate hosts, acquiring *T. solium* cysticerci (larval stage) in their tissue, through the ingestion of *T. solium* eggs shed in the faeces of humans infected with adult tapeworms. Consumption of raw and/or undercooked pork with active *T. solium* cysticerci may result in *T. solium* taeniasis in humans. The presence of porcine cysticercosis impacts negatively on an economy due to costs arising from carcass condemnation and negative impacts on market access and trade of pork.

Although Vietnam is located in a region endemic for *T. solium* [3], the data on porcine cysticercosis are limited. In addition, it is not clear whether porcine cysticercosis is endemic in the country. During 1994 to 2005, the highest reported prevalence of porcine cysticercosis in Vietnam among four carcass-based studies was less than 1% [4]. These estimates were, for the most part, based on studies conducted in commercial slaughterhouses, and therefore do not reflect the prevalence of cysticercosis in pig populations in rural communities that are not processed in commercial slaughterhouses [5]. Of the relatively small number of studies that have quantified the prevalence of porcine cysticercosis in the country, most were conducted prior to 2003 with a focus on the north of Vietnam [4]. To the best of our knowledge, the only study of porcine cysticercosis in the south of Vietnam was carried out in 1994 [6].

Dak Lak Province is located in the Central Highlands in the south of Vietnam. A recent study in the communities of the province demonstrated an apparent prevalence of 1.2% *T. solium* taeniasis [7]. In most communities in the province, pigs are free-roaming and outdoor defaecation is common [8]. These characteristics are conducive for infection and transmission of *T. solium* cysticercosis to pigs. The aim of this study was to estimate the prevalence of cysticercosis in pigs in Dak Lak and to identify household level characteristics associated with *T. solium* porcine cysticercosis.

## Methods

### Study site and sampling

Fieldwork was conducted between June and October 2015 in Krong Nang, M’Drak and Buon Don districts in Dak Lak Province, Vietnam. These districts were chosen as the study sites based on their diverse geographical characteristics. The study sites have been described in detail elsewhere [7]. In brief, M’Drak is located in the east of Dak Lak Province with an average altitude of 400 m to 500 m and has a tropical monsoon climate typical of the Vietnam’s Central Coast. Krong Nang is situated in the north of the province at an altitude of 800 m. Buon Don, situated to the west of the province with an average elevation of 330 m and has a hot and dry climate. The standard of living in this area of Vietnam is generally poor. Open defaecation using outdoor pit latrines is common practice and livestock access to these latrine areas is usually unrestricted. The practice of non-confinement of pigs and cattle is common with slaughter activities often carried out in backyards [4].

A sampling frame listing the name of all villages in the three study districts was obtained from Sub-Department of Animal Health office. Villages eligible for sampling comprised those with more than 1000 pigs, as recorded by the Sub-Department of Animal Health within the Ministry of Agriculture. All eligible villages within each of the three study districts were assigned a number and two numbers were chosen at random to select villages from each district for inclusion in the study. Within each selected village, a list of householder names was obtained from the respective village head person, and each householder name was then coded with a number (the number of households per village ranged from 200 to 300). A sheet of paper was drawn up into squares and each square numbered from 1 to 300. The squares were cut into pieces and placed face-down on a table. The village head was then asked to select between 100 and 140 squares. The number on each selected square identified each household to be sampled. All selected households were visited several days before sampling to obtain consent from participants. Within each district, blood samples were collected from pigs in each of the study households. At the time of each household visit pig owners were asked to complete a questionnaire on the number and type of pigs kept and details of demography, husbandry practices, and diet (Table 1). All questionnaires were conducted in local Vietnamese phraseology and their validity pre-tested on 30 pig owners in another community in Dak Lak Province before application to the field survey. In addition, interviewers were trained before administering the questionnaires. Pigs were selected at random by the member of research group for blood sampling. Pigs that were pregnant or ill, and pigs aged less than 2 months of age were excluded from sampling. At the time of each household visit 10 ml of blood was obtained from the cranial vena cava of each pig into plain blood collection tubes. The blood samples were allowed to clot at ambient temperature prior to centrifugation at 3200× *g* for 5 min to separate serum. Serum was dispensed into 1.5 ml aliquots and stored at -20 °C until use.

**Table 1:** Details of information about general and pig information

Requesting information	Detail
General information	Address of house; number of people per household; presence and type and location of toilet; number of livestock per household; vegetables and crops management (source of water for irrigation, kind of manure for vegetables and crops); coordinate of household, pigsty, toilet and vegetable patch; elevation of household; number of dogs; location of defecation for dogs; efforts to disinfect dog’s faeces; observation of *Taenia* proglottids in dog’s faeces
Pig information	
Demography	Sex; age; breed; number of pigs
Husbandry management	The presence and location of pigsty; whether pig confined or free-roaming; roaming range of pigs; whether pigs eat human faeces; whether pigs have access to human defecation area
Diet management	Drinking water sources for pigs, kind of food for pigs (commercial or handmade bran, scavenging), feeding raw vegetables to pigs or not, vegetables washed before feeding pigs

### Sample size estimation

The aim of this study was to estimate the prevalence of porcine *T. solium* cysticercosis in Dak Lak Province. Based on a previous slaughterhouse based survey by Van De at al. [9], the prevalence of porcine cysticercosis was assumed to be 10%. Assuming 95% certainty that this estimate was within 5% of the actual population prevalence (i.e. cysticercosis prevalence ranged from 5% to 15%) and ignoring the possibility that cysticercosis positive pigs were clustered within households, we estimated that a total of 384 pigs were required to be sampled. We then assumed the average number of pigs eligible for random sampling per household was at least three and an intra-class correlation coefficient for *T. solium* cysticercosis of 0.07 [10] returning a design effect of 1.14. Our revised sample size, accounting for the likelihood that porcine cysticercosis clusters within households, was 384 × 1.14 = 438 for each of the three study districts.

### Laboratory procedures

Pig serum samples were analyzed using the recombinant T24H antigen in enzyme-linked immunoelectrotransfer blot (rT24H-EITB) assay as described by Noh et al. [11] and lentil lectin purified glycoproteins in EITB (LLGP-EITB) assay as previously described by Tsang et al. [12] and Gonzalez et al. [13]. Both the LLGP-EITB and rT24H-EITB assay are immunoblot methods. The LLGP-EITB detects antibodies to one or more of seven lentil lectin purified glycoproteins (LLGPs), namely GP50, GP42, GP24, GP21, GP18, GP14 and GP13 which are present in the soluble fraction of an extract of *T. solium* cysticerci [11]. Reaction to any of these 7 glycoprotein antigens is considered positive. The rT24H-EITB assay detects antibodies against rT24H antigen derived from 24- and 42-kDa glycoproteins of the LLGPs [14].

To ascertain the analytical specificity of the rT24H antigen, we subjected 29 cysticercosis-negative USA pig sera, 12 necropsy-positive *T. solium*-positive Peruvian pig sera and 4 *T. hydatigena* necropsy-positive Vietnamese pig sera to the rT24H-EITB. All USA pig sera and Vietnamese *T. hydatigena* pig sera were negative for the rT24H-EITB, and all Peruvian *T. solium* positive pig sera were positive on the rT24H-EITB. These preliminary results provided the basis of results show that under experimental conditions, the rT24H-EITB do not cross-react to pig sera with *T. hydatigena*.

Individual serum samples were screened in pools of four using the rT24H antigen in EITB assay format to detect the presence of antibodies against *T. solium* cysticerci. The rT24H antigen was utilized in the EITB assay as it offers the best overall diagnostic performance compared with other recombinant or synthetic antigens based on our preliminary data and previous human-based studies [15, 16]. Individual serum sample from each positive pools were then re-screened using rT24H-EITB and LLGP (native antigen of *T. solium* cysticerci) antigens. The LLGP-EITB has been used as the reference standard assay for serological diagnosis of *T. solium* cysticercosis in humans and pigs that has the specificity of 100% and sensitivity between 98–100% [12, 13]. Jayashi et al. [17] when validating the LLGP-EITB for naturally acquired porcine cysticercosis pointed out that the LLGP-EITB achieves optimal sensitivity of 78% (95% CI: 52–94%) and specificity of 76% (95% CI: 66–85%) when the assay reacts to ≥ 3 of 7 LLGP antigens. The diagnostic performance of LLGP-EITB for porcine *T. solium* cysticercosis was evaluated in Peruvian pigs, and the cross-reaction of the assay to *T. hydatigena* was not known [13]. An individual serum sample was considered positive for *T. solium* antibodies if it was positive to both rT24H and native LLGP antigens.

### Statistical analysis

Risk factors for porcine *T. solium* cysticercosis in the communities of Dak Lak Province were identified using logistic regression. In this study, the outcome of interest was a dichotomous variable where households where at least one pig was *T. solium* cysticercosis-positive were assigned a value of 1, and 0 otherwise. The association between each of a set of household-level candidate explanatory variables from the questionnaires and the outcome of interest were tested using unconditional odds ratios and the chi-square test. All explanatory variables associated with the outcome of being *T. solium* cysticercosis positive at an alpha level of < 0.20 using the chi-square test were selected for inclusion in the multivariable model.

A frequentist fixed-effects logistic regression model was developed in which the probability of a household having at least one cysticercosis-positive pig was parameterized as a function of the explanatory variables with significance of the chi-square test at *P* < 0.20, as described above. The significance of each explanatory variable in the model was tested using the chi-square test. Explanatory variables that were not statistically significant were removed from the model one at a time, beginning with the least significant, until the estimated regression coefficients for all the explanatory variables retained in the model were significant at an alpha level of < 0.05.

To account for the hierarchical structure of the data (households within villages) a village-level random effect term (*V*_*i*_), was included in the model as shown in Equation 1. District was not a significant predictor of household level *T. solium* cysticercosis status at the alpha level of 0.05 and was therefore not considered further in the mixed-effects model.

1 $$ \log \kern0.5em \left[\frac{pi}{1- pi}\right]=\kern0.5em {\beta}_0\kern0.5em +\kern0.5em {\beta}_{1\kern0.5em }{x}_{1i}\kern0.5em +\kern0.5em \cdots \kern0.5em +\kern0.5em {\beta}_m{x}_{mi\kern0.5em }\kern0.5em +\kern0.5em {V}_i\kern0.5em +\kern0.5em {\varepsilon}_i $$

Due to the low prevalence of *T. solium* cysticercosis (12 of 1281 pigs were positive) regression coefficients for the mixed-effects logistic regression model were estimated using a Bayesian approach implemented in JAGS [18, 19]. Flat (uninformed) prior distributions were assumed for the intercept *β*_0_ and each of the regression coefficients for the fixed effects *β*_1_ ⋯ *β*_*m*_. The village-level random effect term (*V*_*i*_) was parameterized as having a normal distribution with mean 0 and precision (inverse variance) *τ*.

For each of the Bayesian regression analyses we ran the Markov chain Monte Carlo sampler for 40,000 iterations and discarded the first 1000 ‘burn-in’ samples. Convergence was visually assessed by plotting cumulative path plots for each of the monitored parameters [20, 21] and quantified using the Raftery & Lewis convergence diagnostic [22, 23]. Parallel chains were run using diverse initial values to ensure that convergence was achieved to the same distribution [24]. Posterior sample sizes were determined by running sufficient iterations to ensure that the Monte Carlo standard error of the mean was at least one order of magnitude smaller than the posterior standard deviation for each parameter of interest.

The results of the final mixed-effects logistic regression model are reported in terms of adjusted odds ratios for each explanatory variable. Assuming a causal relationship between a given explanatory variable and porcine cysticercosis, an adjusted odds ratio [and its 95% credible interval (CrI)] of > 1 indicates that, after adjusting for other variables in the model, the explanatory variable increased the risk of a pig being cysticercosis positive. An adjusted odds ratio (and its 95% CrI) of < 1 indicates that exposure to the explanatory variable was protective, and an OR of 1 indicates that the variable was not associated with porcine cysticercosis risk.

Statistical analyses were performed using the packages *R2jags* [25] and *coda* [26] implemented in R version 3.3.0 [27].

## Results

### General description of study population

A total of 1324 pig serum samples were collected in Krong Nang, M’Drak and Buon Don districts in Dak Lak Province. Of these, 1281 samples were eligible for further examination as 43 samples were excluded from analysis due to hemolysis or missing questionnaire information. All 1281 serum samples were screened in pools of 4 using the rT24H-EITB assay, and 10 pool samples were identified as positive. Twelve single samples among the 10 positive pool samples were positive for *T. solium* antibodies using the rT24H-EITB assay and all 12 single samples were positive using LLGP-EITB. The prevalence of *T. solium* cysticercosis in pigs in the study districts was 0.94 (95% CI: 0.51–1.68) per 100 pigs at risk. The 12 positive pigs belonged to 11 households in the three study districts. Of 203 households visited in M’Drak district, 9 (4.43%, 95% CI: 2.17–0.85%) possessed *T. solium* seropositive pigs. Among 70 visited households in Krong Nang district, two (2.8%, 95% CI: 0.49–10.8%) possessed *T. solium* cysticercosis positive pigs, and no seropositive pigs were identified in 135 households in Buon Don District.

The hierarchical structure of the data in this study is shown in Table 2. The 1281 pigs were from 408 households and, within each household, an average of three pigs were sampled (minimum 1; maximum 24).

**Table 2:** Structure of the data from 1281 study pigs from six villages in M’Drak, Buon Don and Krong Nang districts

Level	Number	Number at the next highest level	
		Mean	Range
Districts	3	–	–
Villages	6	2	2
Households	408	68	21–135
Pigs^a^	1281	3	1–24

^a^A total of 1281 pigs recruited in this study. The mean no. of pigs per household was three (range 1–24)

Of the 408 households, 266 (65%, 95% CI: 60–70%) used a pit latrine; however, most of these latrines were of temporary construction, which animals were able to access. A total of 35% (95% CI: 30–40%) of householders responded that their family members practiced outdoor defection; children typically defaecated around the main household building while adults defaecated some distance from the main household building, within the confines of the household property. Seventy-six percent (95% CI: 72–80%) of households had a pigsty and 58% (95% CI: 53–63%) confined their pigs at all times. Free-roaming pigs habitually ranged around the village to seek food and to return to their litters located under stilt housing in the afternoon. Approximately 7% (95% CI: 5–10%) of households used water sourced from lakes, streams or ponds for their pigs. The remaining households used either rainwater or water from wells or pipes. Dogs were kept in 63% (95% CI: 59–68%) of the households that owned pigs (Table 3).

**Table 3:** General description of household data

Characteristic	Frequency	Percentage (95% CI)
Number of households	408	–
District		
M'Drak	203	50 (45–55)
Buon Don	135	33 (28–38)
Krong Nang	70	17 (14–21)
Presence of pit latrine		
Yes	266	65 (60–70)
No	142	35 (30–40)
Presence of pigsty		
Yes	311	76 (72–80)
No	97	24 (20–28)
Are pigs permitted to roam freely?		
Yes	172	42 (37–47)
No	236	58 (53–63)
Source of water for pigs		
Pipe/well/rain water	379	93 (90–95)
Lake/stream/pond	29	7.0 (4.9–10)
Owning dogs		
Yes	259	63 (59–68)
No	149	37 (32–41)
Household observing proglottids in dog faeces		
Yes	113	44 (37–50)
No	146	56 (50–62)
Faeces of dogs treated		
Yes	3	1.1 (0.3–4.6)
No	256	99 (96–99)
Site of dog defecation		
Inside compound	192	74 (68–79)
Outside compound	67	26 (21–32)

Among the 1281 pigs that were sampled, 41% (95% CI: 38–44%) were of local breed (Soc). A total of 27% (95% CI: 24–29%) of the sampled pigs were reported to regularly consume human faeces. A little over half of the pigs were routinely offered raw, unwashed vegetables (57%; 95% CI: 54–59%). Commercial and/or homemade bran was offered to 89% (95% CI: 86–91%) of pigs. The small proportion of pigs that were not supplied bran (11%, 95% CI: 9–12%) were scavenging for the most part (Table 4).

**Table 4:** General description of pig data

Characteristic	Frequency	Percentage (95% CI)
Number of pigs	1281	–
Sex		
Male	536	42 (39–45)
Female	745	58 (55–61)
Age (months)		
< 4	497	39 (36–42)
4–12	665	52 (49–55)
> 12	119	9.3 (7.8–11)
Breed		
Imported breed	522	41 (38–44)
Local breed (Soc)	759	59 (56–62)
Allowed to roam freely		
Yes	406	32 (29–34)
No	875	68 (66–71)
Human coprophagy		
Yes	340	27 (24–29)
No	941	73 (71–76)
Main food		
Commercial/handmade bran	1145	89 (86–91)
Scavenging	136	11 (9.0–12)
Provision of raw, unwashed vegetables^a^		
Yes	723	57 (54–59)
No	555	43 (41–46)

^a^Three cases was excluded from analysis

### Risk factors for porcine *T. solium* cysticercosis

Of the data recorded using the questionnaire, we identified two factors associated with a pig’s likelihood of being *T. solium* cysticercosis positive: (i) frequent coprophagy of human faeces; and (ii) scavenging for food. Estimated regression coefficients for the mixed-effects logistic regression model provided in Table 5. After adjusting for the other explanatory variables in the model, the odds of a household where pigs routinely consumed human faeces being *T. solium* cysticercosis positive was 2.57 (95% CrI: 1.22–4.66) times that of a household where pigs did not consume human faeces. The odds ratio for a household where pigs routinely scavenged for food was 1.98 (95% CrI: 0.55–4.74).

**Table 5:** Risk factors associated with *T. solium* cysticercosis positive in pigs

Explanatory variable	Number of pigs		Regression coefficient (SD)	MC error	Adjusted OR (95% CrI)
	Positive	Total			
Intercept	12	1281	-5.9800 (1.5480)	0.006	–
Coprophagy of human faeces:					
No	3	941	Reference		
Yes	9	340	2.6600 (0.8470)	0.004	2.57 (1.22–4.66)
Kind of food					
Bran	6	1145	Reference		
Scavenging	6	136	2.1400 (1.0320)	0.003	1.98 (0.55–4.74)^a^
Random effects^b^	Estimate	SD			
Village	2.18	5.77			

^a^Interpretation: The odds of *T. solium* cysticercosis positive for pigs that scavenged food was 1.98 (95% credible interval 0.55–4.74) times that of pigs that did not scavenge for food

^b^Variance and standard deviation (SD) of the variance of the village-level random effect

## Discussion

In low-income rural communities of Vietnam, a substantial proportion of the human population practice open defaecation and uncooked pork and beef consumption is relatively common. Allowing pigs to roam freely is a common husbandry practice in the region [4, 5, 9, 28, 29]. Despite all relevant risk factors for cysticercosis being present in each of the communities in this study, the prevalence of *T. solium* cysticercosis was low at 0.94 (95% CI: 0.51–1.68) cases per 100 pigs at risk. A similar, low prevalence of cysticercosis has been observed not only in Dak Lak Province but in other regions of Vietnam [4, 6, 30]. In 1994 Huan, in a cross-sectional study of pigs submitted for slaughter from 12 provinces in the south of Vietnam, reported a prevalence of 0.90 (95% CI: 0.45–1.76) cases per 100 pigs at risk [6]. In three studies carried out in 10 provinces in the north of Vietnam between 1999 and 2003, the prevalence of cysticercosis ranged from 0 to 0.06 cases per 100 pigs at risk [31–33]. In the Province of Bac Ninh in the north of Vietnam, a known foci of *T. solium* in humans, carcass examination of 26 village pigs identified no cases of *T. solium* cysticercosis. Instead, 10 pigs were positive for *T. hydatigena* cysticerci [34]. These findings are in agreement with those of Conlan et al. [35], who reported a low prevalence of *T. solium* cysticercosis of 0.8% in village pigs in Laos with a relatively high prevalence of *T. hydatigena* (22 cases per 100 pigs at risk) and a high prevalence of *T. solium* taeniasis. Conlan et al. [35] hypothesized that *T. hydatigena* is likely to cross-protect pigs from *T. solium* infection.

In Vietnam the prevalence of *T. hydatigena* cysticercosis in pigs has been reported to be high, ranging between 25–38% in the north and the prevalence has been strongly correlated with the presence of *T. hydatigena* infection in dogs [36]. In addition, most of the households that owned pigs in the three study districts also kept dogs and approximately one half of the households owning dogs reported that they had observed proglottids in their dog’s faeces (Table 3). All of the 10 free-roaming village pigs that were backyard slaughtered had *T. hydatigena* cysticerci present in the mesentery, stomach, spleen, and liver (personal observation from fieldwork). It is our inference that the relatively high prevalence of *T. hydatigena* infection in dogs is likely to be a major source of *T. hydatigena* cysticercosis in pigs. If this is true a cautioned approach to cysticercosis control in pigs would be advised if *T. hydatigena* were to be targeted through pig confinement and canine deworming programs. Eliminating or reducing pig exposure to *T. hydatigena* would likely result in an increase in the observed prevalence of *T. solium* in non-compliant free-roaming pigs, providing an increased public health risk to the community.

Among the investigated households that owned pigs, a little under one-quarter did not have a pigsty and most of the pigs that were kept were of the local breed (Table 4). Local breeds are preferred due to their ability to thrive under harsh raising conditions and poor feeding [12]. Allowing pigs to free-roam for food was common practice (Table 3). The odds of a household where pigs routinely scavenged for food being seropositive for *T. solium* cysticercosis was 1.98 (95% CrI: 0.55–4.74) times greater than the odds of a household where pigs were fed commercial and/or homemade bran (Table 5). Similarly, the odds ratio for a household where pigs routinely consumed human faeces being seropositive was 2.57 (95% CrI: 1.22–4.66) times greater than the odds of a household where this practice was not habitual. It is known that risk factors for transmission and circulation of porcine cysticercosis are numerous and may vary in different settings. We found that allowing pigs to free-roam and allowing pigs to routinely consume human faeces were associated with *T. solium* exposure, consistent with other studies [37–39]. Research on the epidemiological characteristics of porcine cysticercosis in Peru [40], Mozambique [41] and Mexico [42] found that older pigs were more likely to show evidence of exposure to *T. solium* compared to younger pigs, an association not identified in this study. Similarly, while studies from Zambia [43], Mexico [44], and Tanzania [45] showed that the prevalence of *T. solium* exposure was higher in male pigs compared with females, this association was not identified in this study. In this study, both of the risk factors identified are modifiable, which means that there exists an opportunity to decrease the prevalence of porcine cysticercosis even further. We propose that a combination of intervention measures including education and public awareness campaigns, strategies to reduce coprophagy among pigs and enhanced meat inspection (particularly backyard slaughtered stock) are likely to have the greatest impact on porcine cysticercosis risk with positive secondary effects on human health [46]. For this to be successful there is a need for commitment and support from local and/or central veterinary and medical health authorities.

## Conclusions

The prevalence of porcine *T. solium* cysticercosis in this study was low at 0.94 (95% CI: 0.51–1.68) cases per 100 pigs at risk, in agreement with other studies conducted throughout Vietnam. Scavenging of food and coprophagy were associated with *T. solium* cysticercosis risk. Both of these characteristics are modifiable providing the opportunity to decrease the prevalence of porcine cysticercosis even further.

## Abbreviations

EITB: enzyme-linked immunoelectrotransfer blot
LLGP: lentil lectin purified glycoprotein
rT24H: recombinant antigen T24H

## References

[1] FAO/WHO. Multicriterial-based Ranking for Risk Management of Food-borne Parasites (2014). Microbiological Risk Assessment Series No 23. Rome: WHO Press.

[2] Donadeu M, Lightowlers MW, Fahrion AS, Kessels J, Donadeu M, Lightowlers MW, et al. *Taenia solium*: WHO endemicity map update. Wkly Epidemiol Rec. 2016;49/50:595–9. 27966846

[3] Aung AK, Spelman DW (2016). *Taenia solium* taeniasis and cysticercosis in southeast Asia. Am J Trop Med Hyg..

[4] Ng-Nguyen D, Stevenson MA, Traub RJ (2017). A systematic review of taeniasis, cysticercosis and trichinellosis in Vietnam. Parasit Vectors..

[5] Huong NT. Taeniasis and cysticercosis in a selected group of inhabitants from a mountainous province in North Vietnam. Antwerpen, Belgium: Prince Leopold Institute of Tropical Medicine; 2006.

[6] Huan L Van. Parasitic helminths in pigs in several southern provinces and preventative measures National Institution of Veterinary Research; 1994. http://luanan.nlv.gov.vn/luanan?a=d&d=TTkFvmnEdmia1994.1.2&e=-%2D-%2D-%2D-vi-20%2D-1%2D-img-txIN-%2D-%2D-%2D-%23. Accessed 10 Nov 2015.

[7] Ng-Nguyen D, Stevenson MA, Dorny P, Gabriël S, Van VT, Nguyen VT (2017). Comparison of a new multiplex real-time PCR with the Kato-Katz thick smear and copro-antigen ELISA for the detection and differentiation of *Taenia* spp. in human stools. PLoS Negl Trop Dis..

[8] Nguyen TH (2009). Evaluation of market opportunities for producing local pigs in Dak Lak. Tay Nguyen J Sci..

[9] Van De N, Le TH, Lien PTH, Eom KS (2014). Current status of taeniasis and cysticercosis in Vietnam. Korean J Parasitol..

[10] Asaava LL, Kitala PM, Gathura PB, Nanyingi MO, Muchemi G, Schelling E (2009). A survey of bovine cysticercosis/human taeniosis in Northern Turkana District, Kenya. Prev Vet Med..

[11] Noh J, Rodriguez S, Lee YM, Handali S, Gonzalez AE, Gilman RH (2014). Recombinant protein- and synthetic peptide-based immunoblot test for diagnosis of neurocysticercosis. J Clin Microbiol..

[12] Tsang VCW, Brand JA, Boyer AE (1989). An enzyme-linked immunoelectrotransfer blot assay and glycoprotein antigens for diagnosing human cysticercosis (*Taenia solium*). J Infect Dis..

[13] Gonzalez AE, Cama V, Gilman RH, Tsang VCW, Pilcher JB, Chavera A (1990). Prevalence and comparison of serologic assays, necropsy, and tongue examination for the diagnosis of porcine cysticercosis in Peru. Am J Trop Med Hyg..

[14] Hancock K, Pattabhi S, Whitfield FW, Yushak ML, Lane WS, Garcia HH (2006). Characterization and cloning of T24, a *Taenia solium* antigen diagnostic for cysticercosis. Mol Biochem Parasitol..

[15] Handali S, Klarman M, Gaspard AN, Noh J, Lee YM, Rodriguez S (2010). Multiantigen print immunoassay for comparison of diagnostic antigens for *Taenia solium* cysticercosis and taeniasis. Clin Vaccine Immunol..

[16] Rodriguez S, Wilkins P, Dorny P (2012). Immunological and molecular diagnosis of cysticercosis. Pathog Glob Health..

[17] Jayashi CM, Gonzalez AE, Castillo Neyra R, Rodríguez S, García HH, Lightowlers MW (2014). Validity of the enzyme-linked immunoelectrotransfer blot (EITB) for naturally acquired porcine cysticercosis. Vet Parasitol.

[18] Kruschke JK (2017). Review of doing Bayesian data analysis: a tutorial with R, JAGS, and Stan (second edition). Clin Neuropsychol.

[19] Plummer M. JAGS Version 3.4.0 user manual. 2013;0–41. http://www.stats.ox.ac.uk/~nicholls/MScMCMC15/jags_user_manual.pdf

[20] Yu B, Mykland P (1998). Looking at Markov samplers through cusum path plots: a simple diagnostic idea. Stat Comput..

[21] Robert CP, Casella G (2004). Monte Carlo Statistical Methods.

[22] Raftery AE, Lewis SM (1992). One long run with diagnostics: implementation strategies for Markov Chain Monte Carlo. Stat Sci..

[23] Raftery EA, Lewis MS. How many iterations in the Gibbs Sampler? In: Benardo J, Berger J, Dawid A, Smith A, editors. Bayesian Stat 4. New York: The Clarendon Press and: Oxford University Press; 1992. p. 763–74.

[24] Gelman A, Gilks W, Richardson S, Spiegelhalter D (1996). Inference and monitoring convergence. Markov Chain Monte Carlo Pract.

[25] Su Y-S, Yajima M. Using R to Run “JAGS.” 2015. https://cran.r-project.org/web/packages/R2jags/R2jags.pdf

[26] Plummer M, Best N, Cowles K, Vines K, Sarkar D, Bates D (2016). Coda: Output Analysis and Diagnostics for MCMC. R News..

[27] Team. R Core. In: R: A Language and Environment for Statistical Computing [Internet]. Vienna, Austria: R Foundation for Statistical Computing; 2017. http://www.r-project.org.

[28] Anh Tuan P, Dung TTK, Nhi VA (2001). Sero-epidemiological investigation of cysticercosis in the southern provinces. J Malar Parasite Dis Control..

[29] Trung DD, Praet N, Cam TDT, Lam BVT, Manh HN, Gabriël S (2013). Assessing the burden of human cysticercosis in Vietnam. Trop Med Int Health..

[30] Willingham AL, Van De N, Doanh NQ, Cong D, Dung TV, Dorny P (2003). Current status of cysticercosis in Vietnam. Southeast Asian J Trop Med Public Health..

[31] Van KP, Luc P (1996). Veterinary Parasitology. Hanoi: Hanoi Agricultural Publishing House.

[32] Doanh NQ, Holland W, Vercruysse J, Van DN (2002). Results of survey on cysticercosis pig in some northern provinces in Vietnam. J Malar Parasite Dis Control..

[33] De N Van, Chau L Van, Son DT, Chuyen LT, Hop NT, Vien HV, et al. *Taenia solium* survey in Hanoi. J Malar Parasite Dis Control. 2004;6:93–99.

[34] Doanh NQ, Kim NT, De NV, Lung NL (2002). Result of survey on taeniasis and cysticercosis humans and pigs in Bac Ninh and Bac Kan provinces. Vet Sci Tech..

[35] Conlan JV, Vongxay K, Khamlome B, Dorny P, Sripa B, Elliot A (2012). A cross-sectional study of *Taenia solium* in a multiple taeniid-endemic region reveals competition may be protective. Am J Trop Med Hyg..

[36] Lan NTK, Quyen NT, Hoat PC (2011). The correlation between the prevalence of the tapeworm *Taenia hydatigena* in dogs and their larvae *Cysticercus tenuicollis* in cattle and pigs - the effect of tapeworm treatment in dogs. Vet Sci Tech..

[37] Ngowi HA, Kassuku AA, Maeda GEM, Boa ME, Carabin H, Willingham AL (2004). Risk factors for the prevalence of porcine cysticercosis in Mbulu District, Tanzania. Vet Parasitol..

[38] Sikasunge CS, Phiri IK, Phiri AM, Dorny P, Siziya S, Willingham AL (2007). Risk factors associated with porcine cysticercosis in selected districts of Eastern and Southern provinces of Zambia. Vet Parasitol..

[39] Pouedet MSR, Zoli AP, Nguekam VL, Assana E, Speybroeck N (2002). Epidemiological survey of swine cysticercosis in two rural communities of West-Cameroon. Vet Parasitol..

[40] Lescano AG, García HH, Gilman RH, Guezala MC, Tsang VCW, Gavidia CM (2007). Swine cysticercosis hotspots surrounding *Taenia solium* tapeworm carriers. Am J Trop Med Hyg..

[41] Pondja A, Neves L, Mlangwa J, Afonso S, Fafetine J, Willingham AL (2010). Prevalence and risk factors of porcine cysticercosis in Angonia District, Mozambique. PLoS Negl Trop Dis..

[42] Sarti Gutierrez E, Schantz PM, Aguilera J, Lopez A (1992). Epidemiologic observations on porcine cysticercosis in a rural community of Michoacan State, Mexico. Vet Parasitol..

[43] Sikasunge CS (2005). The prevalence and transmission risk factors of porcine cysticercosis in eastern and southern provinces of Zambia.

[44] García HH, Gilman RH, Gonzalez AE, Verastegui M, Rodriguez S, Gavidia C (2003). Hyperendemic human and porcine *Taenia solium* infection in Perú. Am J Trop Med Hyg..

[45] Shonyela SM, Mkupasi EM, Sikalizyo SC, Kabemba EM, Ngowi HA, Phiri I (2017). An epidemiological survey of porcine cysticercosis in Nyasa District, Ruvuma Region, Tanzania. Parasite Epidemiol Control..

[46] Gabriël S, Dorny P, Mwape KE, Trevisan C, Braae UC, Magnussen P (2017). Control of *Taenia solium* taeniasis/cysticercosis: The best way forward for sub-Saharan Africa?. Acta Trop..

